# Luteinizing Hormone Regulates Testosterone Production, Leydig Cell Proliferation, Differentiation, and Circadian Rhythm During Spermatogenesis

**DOI:** 10.3390/ijms26083548

**Published:** 2025-04-10

**Authors:** Tian Lei, Yu Yang, Wan-Xi Yang

**Affiliations:** The Sperm Laboratory, College of Life Sciences, Zhejiang University, Hangzhou 310058, China; 3210104827@zju.edu.cn (T.L.); 12207079@zju.edu.cn (Y.Y.)

**Keywords:** luteinizing hormone, Leydig cells, testosterone production, spermatogenesis, male infertility

## Abstract

Male reproductive health, particularly the regulation of spermatogenesis, is controlled by a complex combination of factors, including luteinizing hormone (LH) and its effects on Leydig cells (LCs). LH stimulates testosterone synthesis in LCs, which is critical for maintaining spermatogenesis and male fertility. This review examines the pathways through which LH regulates testosterone production, LC proliferation, differentiation, and circadian rhythm in human and non-human species. In particular, the signaling pathways of luteinizing hormone involved in testosterone production are discussed. Additionally, we explore LH’s role in sperm maturation and quality, emphasizing its clinical implications in treating hypogonadotropic hypogonadism and diagnosing gonadal dysfunctions such as androgen insensitivity syndrome and precocious puberty. Furthermore, the potential of LH in assisted reproductive technologies for improving sperm quality is discussed. By highlighting key molecular mechanisms, this work provides insights into the therapeutic potential of LH in addressing male infertility and conditions of LC dysfunction.

## 1. Introduction

In contemporary society, humans are confronted with a plethora of health issues, with reproductive health concerns garnering increasing attention. A recent study examining sperm quality in Portuguese sperm donors revealed a consistent decline in sperm quality from 2011 to 2018 [1]. Furthermore, 8% to 12% of couples globally are unable to conceive, with male factors accounting for around 50% of cases [2]. Between 1990 and 2017, the rate of age-standardized infertility among men grew by 0.291% year [2]. All of these emphasize the importance of focusing on male reproductive health.

The testis is one of the male sex organs and contains seminiferous tubules and interstitial tissue. Nerves, mast cells, fibrocytes, lymphocytes, LCs, macrophages, and monocytes are all found in the interstitial tissue. The basement membrane separates the seminiferous tubules from interstitial tissues [3]. The synthesis of testosterone by LCs has been demonstrated, and they are crucial for the development of secondary sexual characteristics, gonadal development, and spermatogenesis [4]. Based on their appearance and function, LCs can be classified as either fetal (FLC) or adult (ALC) [5,6].

Spermatogenesis is an extremely important part of the male reproductive process with complex regulatory mechanisms. The process of spermatogenesis is as follows: spermatogonia proliferation, spermatogonia differentiation into spermatocytes, spermatocytes undergo meiosis to produce round spermatids, spermatids undergo metamorphosis to form spermatozoa, and finally spermatozoa release into the lumen of seminiferous tubules [7]. In the human seminiferous tubules, Sertoli cells, peritubular myoid cells, LCs, and the surrounding stroma together form a complex microenvironment that supports spermatogenesis, hormone synthesis, and the maintenance of the blood–testis barrier (reviewed in [8]). During spermatogenesis, some non-coding RNAs, such as miRNAs, piRNAs, and lncRNA, as well as mitochondria, exosomes, and the ubiquitin–proteasome system, have also been found to participate in this precise regulatory process [9,10,11,12,13,14]. In addition, the regulation of spermatogenesis homeostasis is influenced by a number of hormones, including testosterone (T), follicle-stimulating hormone (FSH), and luteinizing hormone (LH) [15]. These hormones regulate spermatogenesis by participating in the regulation of spermatogenic cells or cells around spermatogenic cells [15]. In the regulation of spermatogenesis, each component is very important, and the disorder of any component may cause abnormal spermatogenesis.

LH is one of these hormones that can stimulate LC testosterone release, control LC differentiation, proliferation, and biological rhythm, and ultimately control spermatogenesis [16,17,18]. In addition, LH has been shown to be associated with sperm quality, sperm motility and sperm capacitation [19,20,21]. There is mounting evidence to support the notion that LH is critical to spermatogenesis.

Based on the precise regulation of LH in spermatogenesis, this article reviews the pathways of LH in the process of testosterone production in LCs in the HPG axis, and the specific regulation of LH on the proliferation, differentiation, and biological rhythm of LCs to elucidate the mechanism of LH in the regulation of male spermatogenesis and its role in maintaining spermatogenesis homeostasis. Moreover, considering LH’s pivotal regulatory function and the array of disorders stemming from its aberrant levels, we explore potential clinical applications of LH. By doing so, we aim to offer novel insights and approaches for treating male infertility.

## 2. LH Affects LC Testosterone Secretion and Thus Spermatogenesis

### 2.1. Developmental Processes of FLCs and ALCs

In mice, FLCs can first be recognized at embryonic day 12.5 [22]. After that, their numbers continue to increase, reaching a peak at birth, and then they gradually degenerate [23,24]. The precursors of FLCs may be derived from a variety of cells, such as fibroblasts derived from the mesonephros and fibroblasts derived from the gonadal ridge [25], and it has also been suggested that the precursors of FLCs are derived from coelomic epithelial cells, but this may not be the main source [26]. In addition, some studies have suggested that FLCs may originate from the neural crest, pericytes, vascular-associated cells, or NR5A1-positive precursor cells [27,28]. The increase in the number of FLCs is dependent on the continuous differentiation of progenitor cells rather than mitosis [29].

Many factors jointly regulate this process, such as desert hedgehog (Dhh) and platelet-derived growth factor receptor-α (Pdgfrα). Dhh is derived from Sertoli cells, and its receptor is expressed on LCs and induces differentiation of FLC precursors by maintaining high levels of SF1. PGDF signaling is also required for FLC precursor differentiation [26,30,31]. A number of other factors also regulate the process, including nuclear receptor subfamily 5 group A member 1 (NR5A1), insulin-like growth factor 1 (IGF-1), hepatocyte growth factor (HGF), homeobox gene Aristaless (ARX), platelet-derived growth factor-A (PDGF A), and ovalbumin upstream promoter transcription factor II (COUP-TFII) [32,33]. Testosterone, 5α-dihydrotestosterone (DHT), and insulin-like factor 3 (Insl3) are the main hormones that FLCs produce. Testosterone is produced in a manner similar to that of adults, which synthesize testosterone from cholesterol. However, in mice, FLCs lack the enzyme 17 beta-hydroxysteroid dehydrogenase type 3 (Hsd17b3), which is necessary for ALCs to make testosterone. Furthermore, 11β-hydroxylase (Cyp11b1) and 21-hydroxylase (Cyp21), which have functional similarities with adrenocortical cells, can be expressed by FLCs [34]. Notably, although FLCs contain LH receptor (LHR), which responds to LH, unlike ALCs, their testosterone synthesis is gonadotropin-independent [34].

After birth, the FLCs gradually disappear by degeneration, rather than by apoptosis, and a portion of the FLCs persist in the testicles with ALCs, accounting for about 5% ~ 20%. However, since HSD17B3 and HSD3B6 are absent, their contributions to testosterone generation are smaller or even nil [35,36].

Lymphatic endothelial cells, fibroblasts, and/or pericytes are possible sources of ALCs. Studies have shown that the interstitial compartment’s perivascular and peritubular spindle-shaped cells are implicated in the creation of ALCs [6]. It is uncertain whether the source of ALC stem cells is the same as that of FLCs [37]. ALCs develop from stem LCs (SLCs) and have four developmental stages in rats: SLCs, progenitor LCs (PLCs), immature LCs (ILCs), and ALCs [38]. In rats, the SLCs present a spindle shape do not express LH receptors and do not secrete testosterone [38]. PLCs also have a spindle shape but express 3β-HSD and LHR have a strong ability to produce testosterone [38]. PLCs further differentiate into ILCs, which are round in shape and contain many lipid droplets [38]. Finally, ILCs differentiate into ALCs, intracellular lipid droplets disappear, and the levels of testosterone production and the number of LHR receptors reach their maximum [38]. These phases are shared by ALC restitution in adult testes depleted of LCs as well as the postnatal formation of ALCs [38]. Unlike FLCs, LH and testosterone have strong regulatory effects on the development of ALCs [39]. This will be described in detail below. In addition, mature ALCs can also undergo mitosis to proliferate, which is related to the TGF, IGF1, NGF pathway [34].

### 2.2. Hypothalamic–Pituitary–Gonadal Axis

**Figure 1:**
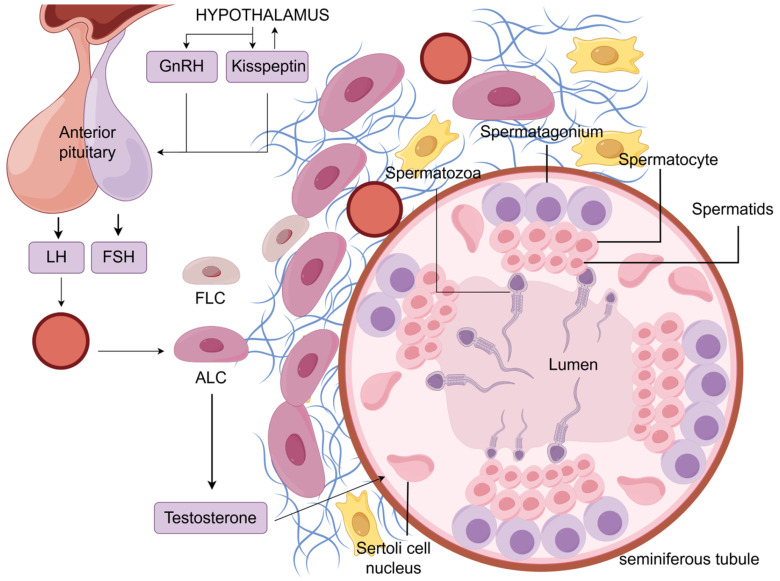
The endocrine regulation of spermatogenesis involving LH. The hypothalamus secretes GnRH and kisspeptin, which act on the pituitary gland, stimulating the synthesis and secretion of LH. LH travels through the bloodstream to the testes, where it acts on ALCs, stimulating the secretion of testosterone. Testosterone enters the seminiferous tubules and acts on Sertoli cells, promoting the differentiation of spermatogonia, spermatocytes, and spermatids into mature spermatozoa, which are released into the lumen of the seminiferous tubules. LCs are present in the testicular interstitium, a loose connective tissue containing various components such as blood vessels, collagen fibers, and LCs (only blood vessels, collagen fibers, and LCs are shown). LCs consist of FLCs and ALCs, but the amount of FLCs is small, and almost no testosterone is produced after birth. Figure support was provided by Figdraw (www.figdraw.com).

The hypothalamic–pituitary–gonadal (HPG) axis is a humoral component of the nervous and endocrine systems, which communicate with each other, and participates in the regulation of testosterone production by LH (Figure 1). The gonadotropin-releasing hormone (GnRH) is one of the most important molecules in the HPG axis; it is a kind of decapeptide molecule produced from hypothalamus neuroendocrine cell synthesis, and it is released into the pituitary gland, where it stimulates luteinizing hormone synthesis and release. LCs have LHR on their surface, which LH binds to initiate a signaling cascade that ultimately results in the production of testosterone [40]. Kisspeptin in the HPG axis is also involved in the synthesis of testosterone. Kisspeptin is a polypeptide synthesized and released by kisspeptin neurons located in the hypothalamus. Kisspeptin promotes gonadotropin secretion primarily by stimulating GnRH secretion from the hypothalamus [41]. In addition, there is also substantial experimental evidence that kisspeptin is likely to directly stimulate pituitary secretion of gonadotropins (reviewed in [42]). Testosterone has a negative feedback effect on hypothalamic GnRH synthesis, but since GnRH neurons do not express androgen receptors, it is hypothesized that the effects of testosterone may be transmitted through other neurons, such as kisspeptin neurons [43].

### 2.3. LH-Stimulated Testosterone Production in LCs

Situated on the surface of LCs, LHRs are members of the G protein-coupled receptor family. Their distinctive feature is their transmembrane domain, which is made up of seven transmembrane helices joined by three extracellular and three intracellular loops [44]. The first identified mutation in LHR was a missense amino acid change, which was found in a family member with familial male precocity, showing a high serum testosterone level and a low serum LH level [45].

In LCs, after LH binds to LHR, the precursor of testosterone, cholesterol, is carried from the cytoplasmic matrix to the interior mitochondrial membrane by transduction bodies such as PKA, steroidogenic acute regulatory protein (StAR), and others [46,47]. CYP11A1 then converts cholesterol to pregnenolone [46,47]. Pregnenolone then leaves the mitochondria and enters the endoplasmic reticulum, where it is mostly converted into progesterone by 3b-hydroxysteroid dehydrogenase (3βHSD) [46,47]. In the endoplasmic reticulum, pregnenolone is metabolized by CYP17 to form dehydroepiandrosterone, progesterone is metabolized by CYP17 to form androstenedione, and androstenedione is also formed from dehydroepiandrosterone by 3βHSD [46,47]. Dehydroepiandrosterone and progesterone are metabolized by 17βHSD to form testosterone [46,47].

**Figure 2:**
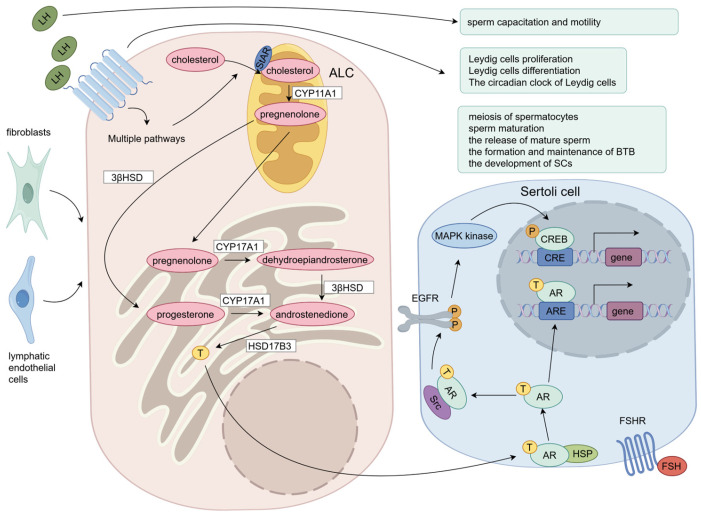
Effects of LH on spermatogenesis, LCs, and sperm cells. LCs originate from fibroblasts or lymphatic endothelial cells. LH can promote testosterone synthesis in LCs, as well as their proliferation, differentiation, and regulation of the biological clock. Upon LH binding to LHR on the surface of ALCs, multiple pathways are initiated, leading to the synthesis of StAR protein located on the mitochondrial membrane. StAR protein facilitates the transfer of cholesterol into mitochondria, where it is converted into pregnenolone by CYP11A1. Pregnenolone undergoes conversion to dehydroepiandrosterone by CYP17A1, which further metabolizes into androstenedione by 3βHSD1 or into progesterone by 3βHSD2 before being converted into androstenedione by CYP17A1. Androstenedione is then synthesized into testosterone by 17βHDS3. Testosterone enters LCs and binds to androgen receptors (ARs), causing dissociation of heat shock proteins. Activated ARs translocate to the nucleus and bind to ARE elements, initiating gene transcription (classical pathway). Activated ARs can also activate MAPK kinase activity through EGFR activation, phosphorylating CREB protein to activate gene transcription (non-classical pathway). Through a series of pathways, this ultimately promotes spermatocyte meiosis, maturation, release, formation, and maintenance of the blood–testis barrier and the development of spermatogonial stem cells. LHR is also present on spermatocytes, suggesting that LH may regulate processes such as sperm energy acquisition, morphological changes, and motility. Figure support was provided by Figdraw (www.figdraw.com).

Testosterone production occurs primarily in the endoplasmic reticulum and mitochondria and involves the catalysis of a variety of enzymes (Figure 2). During testosterone production, cholesterol transfer regulated by StAR proteins is a key step in determining the rate of testosterone production [48]. In 1991, Epstein LF and Orme-Johnson NR found the presence of an LH-induced 30kDa phosphorylated protein in LCs, which was later found to be the result of 37kDa protein cleavage and named steroidogenic acute regulatory protein [49,50]. Recent studies have found that in addition to cholesterol transport, StAR proteins in LCs are implicated in DAG buildup in lipid droplets, which may affect DAG signaling mediated cellular function [51]. Apart from StAR (StARD1), several more START domain proteins have been discovered in mammals, among which StARD4 and StARD5 are found to have a weak effect on cholesterol transfer, while StARD6 has strong activity [52].

LH-regulated testosterone production involves the regulation of multiple signaling pathways, which we discuss in detail below.

### 2.4. Testosterone Regulates Spermatogenesis

LH regulates spermatogenesis by regulating the testosterone secretion of LCs, so how does testosterone regulate spermatogenesis? After being released by LCs, testosterone is partially bound to androgen-binding protein (ABP) for transport [53]. ABP is a glycoprotein secreted by Sertoli cells stimulated by FSH and is present in the testis, epididymis, and liver [53]. In the testis, ABP maintains hormone concentrations, provides a favorable environment for spermatogenesis, and regulates intracellular signals through membrane receptors to affect cell growth, differentiation, and function [53]. When testosterone enters the seminiferous tubules, it binds to androgen receptor (AR) proteins located in peripheral vascular cells such vascular endothelial cells and smooth muscle cells, as well as support cells like Sertoli cells and peritubular myoid cells [54,55]. In the classical pathway, the binding of testosterone to AR leads to its conformational change and detachment of heat shock protein (HSP), and then AR is transferred from the cytoplasm to the nucleus for binding (ARE), thereby expressing activating or inhibiting regulatory proteins to regulate cell activities [56]. In the non-classical signaling pathway, AR is transported to the plasma membrane where it interacts with Src kinase [57]. This ultimately causes the transcription factor CREB to become phosphorylated, which in turn triggers the production of associated proteins [57]. Compared with the classical pathway, the non-classical pathway takes about a few seconds to a few minutes, while the classical pathway takes about 30–45 min [58,59]. Currently, the classical pathway has been found to play a role in neurodevelopment, prostate epithelial homeostasis, bone healing, muscle hypertrophy, reduction in β-amyloid plaques, and reproductive system function (such as spermatogenesis), while the non-classical pathway mainly exists in hippocampal synaptic plasticity, protection of cerebellar granule cells under oxidative stress, migration of prostate cancer cells, anti-apoptosis of neurons, tight junction formation of Sertoli cells in the testis, and alleviation of cardiac ischemia–reperfusion injury [60].

Testosterone, one of the androgens, is essential for spermatogenesis and is found in greater quantity in the testis than in the serum. Testosterone has been found to have a major impact on the meiosis of spermatocytes. According to research by Bartlett JM et al., abnormal spermatogenesis results from acute depression of intratesticular testosterone levels followed by stage-specific degeneration of germ cells, which is mirrored in stage-specific degeneration of pachytene primary spermatocytes at stages VII to VIII of the spermatogenic cycle [61]. In addition, O’Donnell L et al. found that the transition of round spermatids from stages VII to VIII is a crucial T-dependent stage in the spermiogenesis process [62]. Proteomic studies have shown that testosterone may affect sperm meiosis by affecting proteins involved in cell stress and apoptosis, RNA splicing and processing, and DNA repair [63]. In addition to meiosis, testosterone also affects spermatogenesis by influencing the formation and upkeep of the blood–testis barrier, the maturation of sperm, and the growth of Sertoli cells [64,65,66].

In summary, the testosterone secreted by LCs binds to the AR in various cells surrounding spermatogenic cells (primarily Sertoli cells), regulating multiple processes of spermatogenesis through both classical and non-classical pathways (Figure 2).

## 3. LH Regulates Proliferation, Differentiation, and the Circadian Clock of LCs

### 3.1. LH Regulates LC Proliferation

It has been demonstrated that LH regulates LC steroidogenesis in a significant way. Nevertheless, the impact of LH on LC proliferation in the early stages remains unclear, and LH is believed to be less involved in LC proliferation because the period of LC proliferation is independent of the rise in LH concentration, and no appreciable DNA synthesis was seen in LCs stimulated by LH in vitro [67]. After LCs in rats were destroyed by treatment with ethane dimethyl sulphonate (EDS), serum LH levels increased due to a feedback regulatory mechanism; however, PLC proliferation seems to be independent of LH in LC regeneration [17,68]. Nonetheless, subsequent studies have found luteinizing hormone to be indispensable in the proliferation of LCs. When 21-day-old rats were deprived of endogenous LH by LH antiserum, the levels of D-cyclins D3 and proliferating cell nuclear antigen (PCNA), which are strongly linked to cell proliferation, were considerably reduced [16]. Moreover, endogenous LH-deprived mice showed mRNA loss of insulin-like growth factor (IGF-1), a growth factor that has been shown to be strongly linked to the proliferation and development of LCs [16]. In LHR knockout mice, LC counts were much lower [69]. Precocious stimulation with LH alone or in conjunction with FSH increased the mean amount of HSD3B-positive rhesus cells by 20–30-fold, according to a study on pubertal LCs in rhesus monkeys [70]. It also significantly increased their nuclear diameter, which is a hallmark of LC proliferation during early puberty [70]. In the LH-mediated regulation of LC proliferation, ERK1/2 cascade signaling pathway was confirmed to be involved in this process, and neuregulin 1 (NRG1) and collagen IV were also involved [71,72,73]. Stimulation of late pubertal rats with bisphenol B revealed LC proliferation and increased phosphorylation of AKT1/AKT2 and ERK1/2, suggesting that these signaling molecules may be involved in LC proliferation [74]. These results suggest that LH plays an important role in the proliferation of LCs, and the mechanism may involve the ERK and PI3k/AKT signaling pathways.

### 3.2. LH Regulates LC Differentiation

As mentioned above, FLCs, although expressing the LH receptor, are not sensitive to LH stimulation. In the process of ALC formation, there is differentiation from SLCs to PLCs, PLCs to ILCs, and ILCs to ALCs, so what part does LH play in controlling these differentiation processes? LH alone on SLCs isolated from seminiferous tubules did not induce differentiation into PLCs [75]. Moreover, it was discovered that the stem cells developed into cells that could produce testosterone at weeks 2–3 when the spermatogenic tubules were cultured with DHH agonists without LH [37]. These indicate that LH is possibly not necessary for SLCs to differentiate into PLCs. In this process, the testicular microenvironment may participate in the differentiation of SLCs through the leptin-DHH signaling pathway [76]. In adult rats, within 4 weeks following EDS administration, LCs did not develop when LH levels were implanted with testosterone to keep them within the normal range [68]. Numerous 3b-HSD-positive cells were produced by hCG treatment 28 days after EDS delivery, indicating that hCG/LHR is necessary for PLC differentiation [68]. Since rats do not produce CG themselves, LH in rats seems to be important for rat PLC differentiation. In LHR knockout mice, there were no mature LCs and very few progenitor cells [69]. Immature LCs isolated from the testis of 35-day-old rats were treated with LH and found to have a significant increase in steroidogenesis and upregulation of Cyp11a1, Scarb1, Srd5a1, and Cyp17a1, as well as upregulation of biomarkers of mature LCs such as Insl3 [77]. These results indicate that LH can induce ILCs to differentiate into ALCs. However, although LH induces maturation of ILCs, Griffin DK et al. raised the idea that LH may not be an absolute necessity for maturation of LCs in senescent LCs. They found that LHRKO mice treated with testosterone, although highly infertile, exhibited breakthrough spermatogenesis at 12 months of age, suggesting that testosterone can induce LC maturation in aging mice [78]. In conclusion, LH plays an indispensable role in the differentiation of PLCs into ILCs, but it is not necessary for ILC differentiation, although it can promote ILC differentiation into ALCs. In addition, LH may not play a role in the differentiation of SLCs to PLCs.

### 3.3. LH Regulates the Circadian Clock of LCs

The circadian clock serves as an internal timing system present in various tissues and cells, crucial for organisms to adapt to daily changes. The circadian rhythm of cell life activities is regulated by the activators CLOCK (circadian locomotor output cycles protein kaput) and BMAL1 (brain and muscle ARNT-like 1) heterodimers, which recognize E-box motifs to regulate the expression of thousands of genes, including the repressors Period (Per) and cryptochrome (Cry) [79]. These repressors are translated and assembled into repressor complexes to inhibit CLOCK:BMAL1 function, and such feedback regulation leads to a circadian rhythm of cell life activities with a period of about 24 h [79]. LCs also exhibit a circadian clock governing testosterone secretion. After continuous light treatment of growing rats, testis volume decreased, LH receptor gene expression decreased, testosterone production-related gene expression changed, and mitochondrial function of LCs was impaired, suggesting that the stability of circadian rhythm is crucial to the endocrine function of LCs [80]. It has been shown that BMAL1 protein is rhythmically expressed in mouse LCs, and its loss leads to decreased expression of steroidogenic-related genes and decreased testosterone production [81]. Subsequent studies confirmed that clock and steroidogenic gene expression followed circadian rhythms in mouse LCs both in vivo and in vitro [82]. Rhythmic expression of clock-related genes and steroid-related genes was also found in cultured rat and goat LCs in vitro [83,84].

Aging has a significant effect on the rhythm of LCs. Studies have shown that although the endocrine function of rat LCs maintains a circadian rhythm during aging, the robustness and expression level of the rhythm are decreased, and the uptake of cholesterol is also markedly reduced [83]. In rat LCs, aging enhances cGMP signaling and decreases cAMP signaling in LCs, which may affect the process of testosterone secretion [85]. It has also been found that aging leads to decreased circadian rhythm and steroidogenesis through increased ER stress in mice [86].

In the study of the circadian rhythm of testosterone production in LCs, the important role of LH has gradually emerged. In primary cultured LCs, the activation of LHR-cAMP signaling promotes the expression of the StAR gene and changes the expression levels of many clock-related genes, such as the upregulation of Rorb, Dec1/2, and Per1/PER1 and the downregulation of Bmal1 and Rev-erb/b [18]. In rats with artificial hypogonadotropic hypogonadism, decreased secretion of LH and testosterone with loss of circadian rhythm was observed, and decreased Cyp11a1, Cyp17a1, and cAMP with loss of rhythm was observed in LCs [18]. Some genes associated to circadian rhythms, such as Dec1, Csnk1e, Bmal1, Per2, Cry1, Cry2, Rora, Rorb, Rev-erba/b/REV-ERBB, etc., have altered expression levels in LCs, whereas Npas2 and PER1 have decreased levels [18]. Though it was insufficient to preserve the circadian regularity of testosterone production in LCs, the clock gene was still able to sustain rhythmic expression [18]. This indicates that while LCs possess an intrinsic circadian clock, the circadian rhythm of cAMP and testosterone production relies on the reproductive axis’s rhythmicity [18].

Currently, the circadian rhythm of various testicular cells and spermatogenesis has been observed, but the specific mechanisms remain unclear [87]. Further exploration is needed to elucidate the rhythmic mechanism underlying testosterone production by LCs. Although experimental evidence has established that LH is important for regulating the biological rhythm of LC steroidogenesis, further exploration is needed to elucidate the rhythmic mechanism of testosterone production by LCs.

## 4. LH Regulates Testosterone Secretion of LCs Through Multiple Signaling Pathways

LH promotes testosterone production through a variety of pathways, including the cGMP-PKG, MAPK, PI3k/AKT, and calcium signaling pathways, etc. Additionally, excess testosterone can induce feedback regulation of LC testosterone production, reducing testosterone generation to maintain homeostatic levels through signaling pathways like the AMPK pathway and the Slit/Robo signaling pathway.

In LCs, after LH binds to LHR, it generates a large amount of cAMP, inositol triphosphate (IP3), and diacylglycerol (DG), which subsequently phosphorylate the protein kinases PKA and PKC [88,89]. It is important to note that the LH-induced intracellular response in granulosa cells may be different from that in LCs, with LH preferentially acting through the ERK/AKT pathway, while hCG preferentially acts through the cAMP/PKA pathway [90]. In human LCs, although hCG can induce more cAMP generation, hCG is not qualitatively different from LH in terms of cAMP and ERK1/2 activation, and they are equal in activating downstream steroidogenic events, which reflects the difference in the intracellular signal activated by LH between different genders [91]. Subsequently, various transcription factors are further activated through pathways such as MAPK and calcium ions, which stimulate downstream gene expression.

In the process of steroid production, multiple transcription factors are involved in the regulation of StAR transcription, including cAMP response element binding (CREB) protein [92,93]. According to Manna et al., StAR promoter activity, StAR mRNA, StAR protein levels, and cAMP-induced progesterone production were all enhanced by transiently expressing CREB in MA-10 murine LC tumor cells [93]. However, these responses were markedly suppressed in CREB mutants that cannot be phosphorylated [93]. This implies that CREB/CREM family member phosphorylation plays a crucial role in testosterone production and StAR expression. In addition to CREB/M, activator protein 1 (Fos and Jun), C/EBPa and C/EBPb of the C/EBP family, SF-1, and GATA-4 are all involved in the regulation of LH in steroidogenesis through the cAMP signaling pathway. They are phosphorylated by PKA or PKC and interact with each other to promote or inhibit the transcription of the StAR gene (view in [94]). Among them, Fos/Jun and CREB have crosstalk on a single cis element, which has a repressive effect on StAR gene transcription [95]. At the same time, there are multiple factors involved in the post-transcriptional processing of StAR mRNA. LCs were found to harbor both HuR, a nucleoplasmic mRNA shuttle protein that stabilizes transcripts, and Tis11b, a zinc finger RNA instability protein, indicating a potential regulatory role for StAR mRNA stabilization [96,97]. Also, CPE-binding proteins (CPEBs), the mitochondrial A-kinase anchoring protein 121 (AKAP121), and DAX-1 may all play roles in StAR mRNA processing [94,98,99].

**Figure 3:**
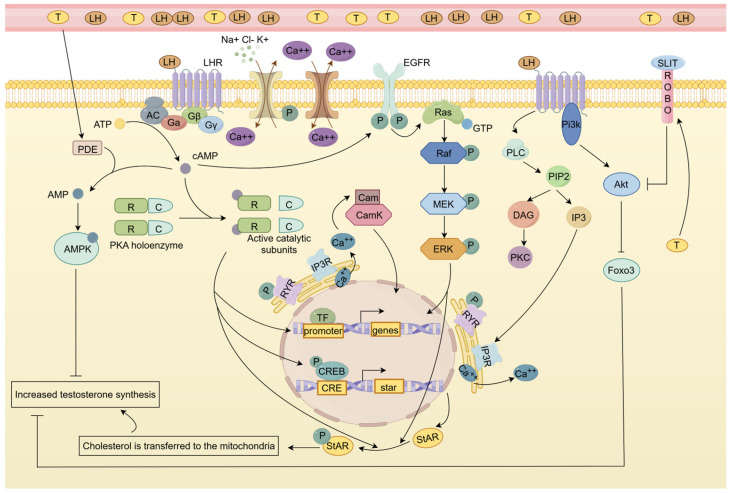
LH regulates testosterone synthesis in LCs through multiple pathways. LH synthesized by the pituitary gland is transported to the testes via the bloodstream, where it binds to the G-protein-coupled receptor LHR on the surface of LCs. This activation leads to the stimulation of adenylate cyclase (AC) and Phospholipase C, resulting in the production of cAMP from ATP and the conversion of PIP2 to IP3 and DAG. cAMP activates PKA, which phosphorylates transcription factors such as CREB, leading to the expression of StAR protein or genes involved in testosterone synthesis. PKA can also directly contribute to the phosphorylation of StAR protein. Additionally, cAMP activates epidermal growth factor receptors (EGFRs) on the LC surface, initiating the MAPK signaling pathway through Ras, Raf, MEK, and ERK phosphorylation, further enhancing gene expression and StAR protein phosphorylation for increased transport capacity. DAG and IP3 activate PKC and IP3Rs on the endoplasmic reticulum, promoting calcium ion release. Calcium ions, in turn, activate CamK, inducing gene expression to facilitate testosterone synthesis. Transcription factor Foxo3 inhibits testosterone synthesis, but LH can suppress Foxo3 inhibition by activating Pi3K and downstream Akt. Excess testosterone promotes PDE-mediated cAMP degradation, activating AMPK to inhibit testosterone synthesis. Testosterone also inhibits its own synthesis by activating ROBO receptors to suppress Akt. These pathways cooperate to maintain dynamic equilibrium in testosterone synthesis. (The production of testosterone in LCs is shown in Figure 2.) Figure support was provided by Figdraw (www.figdraw.com).

In the following section, we will systematically elucidate the key signaling pathways through which LH promotes testosterone synthesis in LCs, with the regulatory network illustrated in Figure 3.

### 4.1. EGFR/MAPK Signaling Pathway Is Involved in LH-Induced LC Testosterone Synthesis

The mitogen-activating pathway, or MAPK, is a crucial signal pathway involved in cell proliferation, differentiation, apoptosis, and stress response in both healthy and pathological settings. It is a part of the eukaryotic signal transduction network. Hirakawa T et al. shown that LHR receptors can trigger LC ERK1/2 phosphorylation in testis LCs, which is mediated by cAMP and PKA [100]. Subsequently, further studies revealed that LHR activated ERK1/2 phosphorylation through growth factor receptor (EGFR) phosphorylation [101,102]. In mouse LCs, LH induces EGFR transactivation in two pathways, MMP-mediated and cAMP-mediated [103]. But in LCs, MMP inhibition had no discernible impact on the synthesis of testosterone, suggesting that the MMP-mediated pathway is not thought to be involved in steroidogenesis [104]. In mouse LCs, it was found that in a short period (<60 min), rapid EGFR signaling activates the ERK signaling pathway and induces phosphorylation of intracellular STAR protein to promote steroidogenesis; however, with prolonged hCG treatment (>2 h), the EGFR/ERK signaling pathway was no longer involved in the steroidogenesis of LCs, and cAMP maintained testosterone production in LCs by promoting StAR transcription [105]. Reduced LH-induced steroid synthesis and higher expression of StAR protein were seen in rat primary LCs and tumor LC lines preincubated with the mitogen-activated protein kinase (MEK) inhibitors U0126 and PD98059 [106]. This suggests an important role for EGFR/LHR interaction in steroidogenic homeostasis in LCs of the testis. Furthermore, in the LCs of Mek1f/f, Mek2−/−, and Cre+ adult mice, the expression of several genes encoding androgen metabolism enzymes (Srda1 and Dhrs9) was upregulated, while the expression of many genes involved in testosterone synthesis (StAR, Hsd3b6, Cyp17a1, and Hsd17b3) was decreased, resulting in a net decrease in steroidogenesis [107]. This also demonstrated the regulatory role of ERK signaling in testosterone secretion in LCs. It has recently been shown that ERK5 knockdown decreases steroidogenesis and decreases the expression of StAR and Nr4a1 (nuclear receptor subfamily 4, group A, member 1, also known as NUR77/NGFI-B) in human chorionic gonadotrophin (HCG)-treated MA-10 LCs, through phosphorylation of MEF2 [108]. Since HCG and LH share the same receptor, whether LH can regulate testosterone production through ERK5 signaling deserves further investigation.

In conclusion, LH regulates testosterone synthesis in LCs in a time-dependent (mainly short-term) manner through activating cAMP/PKA and EGFR-ERK1/2/ERK5 signaling pathways, while ERK5 may affect steroid synthetase expression through the MEF2-Nr4a1 pathway.

### 4.2. Calcium Signaling Pathway Is Involved in LH-Induced LC Testosterone Synthesis

Numerous processes are mediated by calcium signaling and its interaction network, such as embryonic development, gene expression, synaptic transmission, excitation–contraction coupling, stimulus–secretion coupling, and induction of synaptic plasticity [109]. Calcium is also involved in the regulation of LH-induced steroidogenesis in LCs. In LCs, the influx pathway of LH-stimulated changes in calcium concentration is associated with T-type Ca^2+^ channels at the interstitial membrane, whereas the ER release pathway is dependent on the IP3 receptor (IP3R) and the ryanodine receptor (RyR) [110,111]. LH stimulation of intracellular calcium concentration depends on two pathways: the cAMP-dependent and cAMP-independent pathways. Significant changes in testosterone and [Ca^2+^]i were found in rat LCs upon stimulation with low concentrations of sheep LH treatment, whereas no increase in cyclic AMP was detected [112]. Therefore, at low concentrations of LH (below 0.1 pg /mL), [Ca^2+^] may be a regulator of steroidogenesis rather than cyclic AMP. However, when stimulated by a high level of sheep LH, the concentration of calcium ion and cAMP increased, and cAMP could stimulate the increase in calcium ion concentration [112]. However, due to species differences, whether the conclusions obtained by treating rat LCs with sheep LH are consistent with the real response in vivo is worthy of further investigation. In addition, cAMP-activated PKA and PKC can also induce calcium elevation, and the induction is mainly by PKA [113]. The increase in calcium concentration in the cytoplasm depends on two pathways: calcium release from the endoplasmic reticulum and calcium influx from the extracellular matrix. Calcium ions in the testicular stromal cells can be transported to the mitochondria and promote the formation of NADH and NADPH, thus contributing to the mitochondrial testosterone production process [110]. In addition, calcium regulates steroidogenesis by activating the early orphan nuclear receptor NUR77 via calmodulin-dependent protein kinase (CaMK), which in turn promotes the expression of StAR [114,115]. Among various CAMKs, CAMKI has the most significant effect on LC steroidogenesis (reviewed in [116]). One such mechanism is that the co-activator p300 is recruited to the nur77 promoter to activate its expression through AP1, CREB, and MEF2 transcription factors [117].

However, Pandey AK et al. found that after blocking L-type calcium channels in MA-10 mouse LCs, subthreshold cAMP induction (which could not induce StAR expression under normal conditions) significantly increased StAR protein expression and progesterone production, which may be related to the downregulation of DAX-1 expression [118]. The above phenomenon was not produced when L-type calcium channels were blocked alone [118]. This suggests that the blockade of calcium channels “compensates” the sensitivity of LCs to cAMP and that calcium has a complex bidirectional regulation of testosterone production. In hamster LCs, the large-conductance Ca^2+^-activated K+-channel (BKCa) was discovered [119]. It was discovered that blocking this receptor raised testosterone levels, indicating that it might control steroidogenesis by lowering calcium influx through membrane hyperpolarization [119]. It has recently been discovered that LH can support the regulation of mitochondrial membrane potential, STAR processing, and testosterone production by SH3 and cysteine-rich protein 3 (STAC3) [120]. Since STAC3 plays a key role in calcium signaling, it may act as an important factor in the calcium signaling pathway regulating testosterone synthesis.

In addition to calcium ions, other ions are also involved in the regulation of LC steroid production by LH, including chloride ions. When LCs were cultured in medium without chloride, testosterone was found to be increased, whereas chloride deprivation significantly increased StAR protein levels in cells incubated with (Bu) 2cAMP, suggesting a regulatory effect of chloride on LCs, possibly through StAR protein [121]. And it was also found that chloride channel blocker 4, 4′-diisothiocyanate-stilbene-2, and 2′-disulfonic acid (DIDS) inhibited the LH-stimulated steroidogenesis of LCs but had no effect on dibutyryl cyclic adenosine monophosphate (dbc AMP)-stimulated steroidogenesis [122]. Therefore, it can be inferred that LH-stimulated chloride efflux can affect the level of cAMP, which in turn affects the synthesis of STAR protein to regulate steroidogenesis [122]. Even though chloride ions have been shown to be crucial for controlling steroidogenesis, more research is still needed to fully understand the ion channels and underlying processes.

In summary, LH bidirectionally regulates testosterone synthesis in LCs via calcium signaling (Figure 3), mainly through the cAMP-CamK-Nur77 pathway, and calcium channel blockade may enhance cAMP sensitivity.

### 4.3. cGMP-PKG Signaling Pathway Has a Bidirectional Effect on LH-Induced Testosterone Production

In addition to cAMP, cGMP may also be involved in the regulation of the LH steroid secretion of LCs, but it is worth noting that it has a biphasic effect. The intracellular messenger cGMP is generated by guanylate cyclase (mGC) and nitric oxide (NO)-dependent soluble guanylate cyclase (sGC) [123]. It regulates effector function either directly or via cGMP-dependent protein kinase (PKG) [123]. In LCs, nitric oxide is produced by the enzyme nitric oxide synthase (NOS), which is present in three isoforms: endothelial (NOS3), neural (NOS1), and inducible (NOS2). The NO-cGTP signaling pathway has biphasic effects on steroidogenesis in mouse LCs: at low concentrations, NO promotes steroidogenesis, while at high concentrations, NO acts as an inhibitory effect [124].

Andric et al. found that in the rat testis, cGMP stimulates testosterone production in LCs through the phosphorylation of StAR protein, which promotes cholesterol transport to mitochondria [125]. Recent studies have shown that senescent LCs are characterized by elevated cGMP, which leads to decreased ATP production as well as altered mitochondrial function [126]. Subsequent downregulation of NO-cGMP signaling improved mitochondrial dynamics and increased the ATP and testosterone levels of LCs [126]. Andric et al. found that prolonged treatment of intact rats with testosterone enanthate resulted in a significant decrease in serum LH and significantly reduced the ability of purified LCs to produce testosterone in vitro, and further experiments showed that testosterone inhibited LC testosterone production by increasing NOS2 synthesis and decreasing cGMP production [127]. This suggests that although the cGMP signaling pathway does not regulate steroidogenesis through the LH receptor pathway, it may be involved in LH-mediated steroidogenesis by the autocrine regulatory mechanism.

These results suggest that cGMP regulates testosterone synthesis in LCs in a concentration-dependent manner (enhanced at low concentration and inhibited at high concentration) through a mechanism involving StAR phosphorylation and mitochondrial function. An abnormal cGMP-NOS2 axis during aging or testosterone-negative feedback leads to dys-synthesis, suggesting that it acts as an indirect LH pathway to affect steroidogenesis through an autocrine mechanism.

### 4.4. AMPK Signaling Pathway and Slit/Robo Pathway Have Feedback Regulatory Effects on LH-Induced Testosterone Production

In the regulation of LC steroidogenesis by LH, multiple pathways maintain steroid homeostasis through feedback regulation mechanisms. In LCs, phosphodiesterase (PDE) dephosphorylates cAMP and cGMP, resulting in reduced steroid production. PDE5 inhibition was found to increase cGMP and testosterone levels in LCs [125]. In LCs of PDE8A knockout mice, a fourfold increase in their LH-stimulated testosterone production sensitivity was detected [128]. However, in wild-type mice, treatment with 3-isobutyl-1-methylxanthine, a substance that inhibits all cAMP PDEs except PDE8A, resulted in just a slight increase in LH-induced testosterone synthesis; in PDEA8 knockout animals, this increase was more noticeable [128]. This suggests that PDE8A acts in concert with the remaining PDEs to regulate AMP levels in LCs. In addition, it was found that PDE8B is also involved in LH-induced steroid production and that simultaneous inhibition of PDE8s and PDE4s is required for a substantial increase in the PKA-mediated phosphorylation of several proteins related to the regulation of steroid production, including increased expression of the StAR protein [129].

In LCs, PDE converts cAMP to AMP, and AMP activates AMP-activated protein kinase (AMPK), which in turn regulates related transcription factors through the AMPK signaling pathway to regulate steroid production. In knockout mice for AMPK catalytic subunit alpha 1, high levels of testosterone and increases in 3βHSD, CYP17, and StAR proteins are observed, which shows the inhibiting effect of AMPK on steroidogenesis [130]. Further studies showed that the inhibitory effect of AMPK on testosterone production may be through decreasing the expression of the steroidogenic activators c-jun, NUR77, and CREB or increasing the expression of steroidogenic inhibitors such as c-Fos and NR0B1 [131]. Meanwhile, testosterone induced the production of AMPK, which is induced by the degradation of cAMP by PDE, demonstrating the role of AMPK signaling in the regulation of steroid homeostasis. Recently, in the study of the global phosphoproteomic profile of MA-10 LCs in response to Fsk (stimulation of steroidogenesis) and AICAR-mediated AMPK activation (repression of steroidogenesis), it was found that the AMPK signaling pathway downregulates the transcription of StAR and nur77 and affects the phosphorylation of a variety of steroidogenic-related proteins [132]. Notably, AMPK increases CREB levels in cells, and since cleavage of CREB exons results in a switch from activator to repressor to germ cells, it is plausible that active AMPK influences the utilization of CREB exons, and in this case, CREB may prevent the expression of steroidogenic genes [132,133].

Recent studies have shown that the regulation of steroid synthesis in mouse LCs is likewise mediated by Slit/Robo signaling. The homologous single-channel transmembrane receptors of the ring (ROBO) family are bound by secreted glycoproteins known as SLIT ligands, which play a role in controlling cell adhesion, proliferation, and survival in a range of tissues [134]. Slit ligands exist in mouse LCs, and testosterone can increase their expression. Reduced LHR and steroidogenic gene expression, CREB phosphorylation, AKT activity, LH signaling, and testosterone levels were all observed in LCs treated with SLIT in vitro [135]. In the meantime, StAR expression and testosterone synthesis in LCs were observed to be elevated in mice lacking a gene [135]. These findings imply that, in order to preserve the equilibrium of steroids in the body, Slit/Robo signaling may control LH-induced steroid production through feedback regulation. More research is required to elucidate the regulatory mechanism of Slit/Robo, as there are currently only a few studies on the regulatory influence of this protein on the generation of testosterone in LCs.

In summary, LH has been found to maintain steroid homeostasis in LCs through feedback mechanisms of the PDE-cAMP/cGMP-AMPK axis (downregulating StAR/NUR77) and Slit/Robo pathway (inhibiting LHR signaling), where AMPK plays a central inhibitory role through transcription factor reprogramming, while Slit/Robo may serve as a novel testosterone-negative regulator.

## 5. Clinical Application of LH: Treatment of HH, Diagnosis of Gonadal-Related Diseases, and Monitoring of Sperm Quality

### 5.1. Treatment of Hypogonadotropic Hypogonadism (HH): By Rescuing the Broken HPG Axis

Given LH’s pivotal role in LC differentiation, proliferation, testosterone production, spermatogenesis, and sperm capacitation, it holds significant clinical relevance in disease treatment. One notable application is its use in treating hypogonadotropic hypogonadism (HH). The clinical manifestations are often a high degree of infertility, lack of prominent secondary sexual characteristics, and gonadal dysgenesis [136]. There are many causes of HH, including congenital factors, such as isolated GnRH deficiency(IGD), LHR receptor mutation, LHB or FSHB mutation, combined pituitary hormone deficiency, genetic syndrome, etc. [137,138]. There are also acquired factors, such as tumor, lymphocytic infiltration disease, and iatrogenic diseases [137]. Some of these patients with isolated GnRH deficiency present with anosmia, which is known as Kaman syndrome [139].

For the treatment of HH, gonadotropin replacement therapy (GnRH, FSH, LH, or a mixture of LH and FSH) can be used to promote gonadal development and improve fertility [137]. However, because LH is expensive, hCG is often used in clinical practice to replace the testosterone-promoting effect of LH. Because LH and hCG have different half-lives and pharmacokinetics, these differences need to be considered when considering the dose to be used [140]. Regarding their efficacy, studies have shown that their results are highly variable, less effective in individuals lacking mini-puberty (the first six months of neonatal life when the HGP axis is most active), and more effective in individuals with partial pubertal development, indicating the importance of the period of gonadotropin treatment for congenital HH [141].

Several experiments have demonstrated the important role of LH or HCG in gonadal development and hormone production in neonates and secondary sexual characteristics development in adolescents, sex hormone production, and spermatogenesis in adult infertility. In one-year-old neonates with congenital hypogonadotropic hypogonadism (CHH), subcutaneous injection of recombinant LH (rLH) and recombinant FSH (rFSH) led to a notable increase in testicular length from 1.6 cm to 2.4 cm, along with a 170% rise in testicular volume [142]. Importantly, no significant adverse effects were observed [142]. In another study, pump-administered rLH and rFSH to two neonates for several months showed therapeutic effects with penile growth, increased testicular volume, and endogenous testosterone production [143]. A recent study of 65 newborns with CHH who received rhLH and rhFSH treatment by either pump or injection showed that both methods significantly improved the development of mini-puberty but that the injection group experienced a significantly greater increase in testosterone levels and penis length than the pump group, and there were no other differences [144]. Numerous studies have demonstrated the good efficacy of treating gonadal development in adolescents with HH. For example, when HCG and rFSH were administered to adolescents with HH for more than four years until testicular development stopped, it was found that testicular volume, sperm concentration, and quality of life were significantly improved in both pre-pubertal and non-pubertal adolescents with or without testosterone treatment, greatly reducing the impact of gonadal dysgenesis [145].

In adult infertility, in addition to T therapy and pulsatile GnRH therapy, gonadotropin therapy is also commonly used [146]. The usual dose of HCG is 75 to 225IU two to three times a week, often adjusted according to the amount of testosterone in the serum [146]. For most patients, injections of 1500 IU of hCG twice weekly can be used to produce normal testosterone levels, but for some patients 10,000 IU may be required [147]. The use of hCG alone can induce semen production in patients with CHH, but if severe oligospermia or azoospermia persists after 3 to 4 months of treatment alone, it is necessary to add 150–225 IU of FSH three times a week for 6 to 24 months, resulting in testicular growth in almost all patients and in 80% to 90% of patients without cryptorchidism [147].

A substantial amount of clinical research evidence has already proven the effectiveness of hCG in improving testosterone production by LCs. However, the study by Park et al. found that hCG can induce ER stress by activating the UPR pathway, which plays a significant role in steroidogenic enzyme expression [148]. Moreover, high-dose hCG exposure can lead to ER stress-mediated apoptosis in mLTC-1 cells and mouse testes [148]. This has prompted us to pay more attention to the dosage range and duration of use of hCG in clinical applications.

### 5.2. LH Level Can Be Used in the Diagnosis of Androgen Insensitivity Syndrome and Precocious Puberty

As mentioned above, the HPG axis and its involved hormones, such as LH, FSH, and testosterone, play an important role in maintaining male characteristics and spermatogenesis. Abnormalities in this axis often lead to a variety of diseases, among which LH level can be used as one of the bases for disease diagnosis.

Androgen insensitivity syndrome, a disorder associated with the X chromosome due to abnormalities in the androgen receptor (AR) gene, is the most common cause of neutral developmental disorders in 46, XY individuals [149]. Among them, complete type shows normal female external genitalia, partial type shows dysplastic male external genitalia, and mild type shows infertility and gynecomastia [150]. The endocrine features of AIS are characterized by elevated serum androgens and serum LH due to impaired negative feedback regulatory mechanisms of the pituitary gland [150]. A study including 39 Chinese patients with AIS revealed that the basal LH level was about 23 and 17 times higher in CAIS (27.39 ± 3.18 IU/L) and PAIS (18.86 ± 2.25 IU/L) than in healthy children [151]. The peak LH levels were also significantly higher in both conditions than in healthy children [151]. The elevated level of LH can be used as one of the bases for the diagnosis of AIS. AIS is currently tested in children by a human chorionic gonadotropin (hCG) stimulation test and by measuring serum androstenedione, testosterone, and DHT after 72 h. In adults, by measuring elevated/normal total testosterone and increased luteinizing hormone (revealing an overview of androgen resistance), FSH and statin B levels may be within the normal range.

In addition, precocious puberty is also one of the diseases caused by abnormal sex hormones. Precocious puberty refers to the premature development of secondary sexual characteristics, which is manifested as the onset of puberty two standard deviations earlier than the population norm, that is, breast or pubic hair development in girls before the age of 8 years and testicular or penile enlargement in boys before the age of 9 years [152]. The two types of precocious puberty in males are called peripheral precocious puberty, which results from aberrant adrenal and/or testicular dysfunction, and central precocious puberty, which results from premature activation of the hypothalamic–pituitary–gonadal axis. It is especially crucial to identify premature puberty in time since it can result in earlier bone aging and be brought on by organic lesions [153]. LH is the best marker of the onset of puberty, and the detection of LH level can be used as one of the bases for judging precocious puberty [154]. Individuals with central precocious puberty have significantly higher LH levels than normal individuals. In peripheral precocious puberty, the marked excess of LH may be caused by HCG tumors or by defects in activating LHCG receptors (testotoxicosis), which lead to LC proliferation and precocious puberty [152]. LH concentrations can also be used to assess the effect of treatment. If the LH peak value of GnRH stimulation is lower than 2.5–4.5 mL/U, the HPG axis is successfully suppressed [155]. A study also found that first-voided urinary LH level measurement can adequately assess precocious puberty treatment with a cutoff value for LH of 1.01 mIU/mL [156].

### 5.3. Sperm Quality Monitoring and Regulation

Differences in the morphology and function of sperm affect their ability to fertilize, so it is particularly important to evaluate the quality of sperm, especially in assisted reproductive technology. Studies on sperm quality are mainly concerned with sperm morphology, sperm swimming ability, sperm bioenergetics, and sperm capacitation, which are very important for sperm successful fertilization [157]. The detection methods of sperm quality mainly involve microscope observation, computer-aided system evaluation, extracellular flux analysis, image flow cytometry, etc. [157]. In addition, many studies have shown a strong correlation between LH and sperm quality, and LH may be used as one of the auxiliary means for sperm quality detection and regulation.

The previous discussion emphasized the importance of the LH and HPG axis in reproduction, and it can be said that the level of LH is extremely important for the normal development of spermatogenesis. In terms of sperm concentration and motility, an earlier study comparing normospermic and oligospermic men found that oligospermic individuals exhibited reduced plasma sperm concentration and motility, with blood LH concentration showing significant associations with sperm cell concentration (*p* < 0.05) and motility (*p* < 0.02) [19]. This suggests that blood LH concentration may affect sperm concentration as well as sperm motility. Also, after comparing the circulating sex hormones and sperm quality in 338 men, only LH was found to be associated with progressive sperm motility [158]. A study on Holstein bulls also showed that sperm density and fresh sperm motility were associated with LHR genotype [159]. In LHR knockout mice, a significant reduction in sperm morphology, motility, and fertility rate was found, which could only be partially rescued by T treatment, indicating the irreversibly important role of LH in maintaining sperm quality [160].

In sperm capacitation and sperm bioenergetics, the researchers found that a functional LH receptor is present in sperm, located in the head of the sperm, a region that experiences morphological and biochemical changes during capacitation, and its activation leads to an increase in cAMP and PKA [20]. It is speculated that the LHR in sperm may be stimulated by LH from the serum or the female reproductive tract and play a role in sperm capacitation, metamorphosis, and motility. Since LHR receptors are present in the epididymis, and the acquisition of sperm motility occurs during sperm maturation in the epididymis, it is suggested that there is a biochemical process of LH-regulated sperm motility in the epididymis [158,161]. Healthy human sperm treated in vitro with LH did not alter the acrosome reaction, but it did cause alterations in sperm capacitation associated with the phosphorylation of the protein tyrosine, a reduction in sperm rapid progressive motility, and a rise in intracellular calcium concentration [21]. The effects occurred at concentrations of 0.5 and 1 mg/mL of hormone, similar to the peak concentrations achieved during the preovulatory LH elevation, indicating that LH is essential for sperm capacitation in the female reproductive tract, as it occurs there [21,162].

These research findings collectively suggest that LH may play a role in sperm capacitation, motility, and maturation, potentially serving as a supplementary approach in assisted reproduction through its ex vivo manipulation of sperm. Furthermore, the significant correlation observed between LH concentration in blood and sperm quality suggests that LH concentration testing could also serve as a method for assessing sperm quality.

## 6. Conclusions and Perspectives

In summary, this study systematically revealed the comprehensive mechanism of LH in regulating testosterone synthesis, proliferation, differentiation, and sperm function in LCs through multiple signaling pathways (Table 1).

The homeostasis of testosterone production in LCs plays a crucial role in regulating spermatogenesis. As the central signaling molecule for testosterone synthesis in LCs, LH maintains testosterone production homeostasis through various pathways. Among these pathways, cAMP/PKA, calcium, EGFR/MAPK, cGMP-PKG, and other pathways play an important positive response role, while AMPK and Slit/Robo maintain the homeostasis of testosterone production through a negative feedback regulation mechanism. Additionally, LH is involved in processes such as LC proliferation, differentiation, and circadian rhythm regulation, making it a key factor in maintaining LC function and population. Currently, LH is utilized in clinical practice for the treatment of conditions such as hypogonadotropic hypogonadism, and it is also extensively employed in the diagnosis of disorders associated with gonadal function, including androgen insensitivity syndrome and precocious puberty. Moreover, many studies have shown that LH may affect sperm concentration, sperm motility, and sperm capacitation, although the underlying mechanisms are not fully understood. Therefore, LH holds potential as an adjunctive hormone for in vitro reproduction and shows promise in the treatment of male infertility conditions such as asthenospermia, male gonadal dysgenesis, or abnormal spermatogenesis resulting from LC dysfunction. In addition, although LH plays an important role in the maturation and function of LCs, the precise regulatory mechanisms of LH in LC proliferation, differentiation, and circadian rhythm remain unclear. Identifying the regulatory molecules and targets through transcriptomic and proteomic approaches may offer new avenues for treating male gonadal dysgenesis characterized by low testosterone levels due to LC abnormalities.

## Figures and Tables

**Table 1:** Functional roles of LH in various processes of spermatogenesis.

Role	Signaling Pathway	Targets	Species/Cell Lines	References
Testosterone production	The ERK pathway	StAR, Hsd3b6, Cyp17a1, and Hsd17b3	Rat	[100,101,102,106]
	The JNK pathway	c-Jun	Rat	[95]
	The ERK5 pathway	StAR and Nr4a1	MA-10 LCs	[108]
	The calcium signaling pathway	STAC3, CaMK, Dax-1	MA-10 LCs, hamster	[110,111,118,119]
	The cGMP-PKG pathway	StAR	Mouse, rat	[124,125,126,127]
	The PI3k/AKT pathway	FOXO3	LC lines R2C	[163]
	The AMPK signaling pathway	3βHSD, CYP17, and STAR	Mouse, MA-10 LCs	[128,129,130,131,132,133]
	The Slit/Robo pathway	CREB, AKT	Mouse	[134,135]
LCs proliferation	The ERK pathway	PCNA, D-cyclins D3	Mouse, rat	[71,72,73,74]
	The PI3k/AKT pathway		Mouse, rat	[74]
LCs differentiation	Unknown	Scarb1, Cyp11a1, Cyp17a1, and Srd5a1, Insl3	Mouse, rat	[68,69,77,78]
The circadian clock of LCs	Unknown	Per1/PER1, Dec1/2 and Rorb et al.	Rat	[18]
Sperm motility	Unknown	Unknown	Human, Holstein bulls	[19,159]
sperm concentration	Unknown	Unknown	Human, Holstein bulls, rat	[19,159]
Sperm morphology	Unknown	Unknown	Rat	[160]
Sperm capacity	The cAMP/PKA pathway	Unknown	Rat, human	[20,21,162]

## Data Availability

Not applicable.
